# Ganodermanontriol regulates tumor-associated M2 macrophage polarization in gastric cancer

**DOI:** 10.18632/aging.205434

**Published:** 2024-01-19

**Authors:** Likang Zhang, Pinghui Shi, Peng Jin, Zhenwei Chen, Biwen Hu, Chenxi Cao, Xiaoguang Wang, Jian Sheng

**Affiliations:** 1 Department of Gastroenterology, The Fifth Affiliated Hospital of Kunming Medical University, Gejiu Peoples Hospital, Gejiu, Yunnan Province, P.R. China; 2 Department of Pharmacy, Suining Branch of the Hospital Affiliated to Xuzhou Medical University, Suining, P.R. China; 3 Department of Colorectal Surgery, The Second Affiliated Hospital of Jiaxing University, Jiaxing, Zhejiang Province, P.R. China; 4 Department of Surgery, The Second Affiliated Hospital of Jiaxing University, Jiaxing, Zhejiang Province, P.R. China

**Keywords:** gastric cancer, M2 macrophage, ganodermanontriol, microenvironment, STAT6

## Abstract

Aim: We focused on investigating the role and mechanism of ganodermanontriol (GAN) in regulating the M2 polarization of tumor-associated macrophages in the gastric cancer microenvironment.

Methods: M2 polarization of RAW264.7 macrophages was induced by IL-4 or co-culture with MFC, and the expression levels of M1 macrophage markers (TNF-α, IFN-γ, IL-1β) and M2 macrophage markers (IL-10, TGF-β, Arg-1) were detected by enzyme-linked immunosorbed assay (ELISA). The protein expression was assayed by Western-Blotting. For *in vitro* experiments, a tumor-bearing mouse model was established, with which the CD206 level was detected by histochemistry, and the binding mode between GAN and STAT6 was simulated through molecular dynamics.

Results: Both IL-4 and MFC could induce the M2 polarization of macrophages. GAN could inhibit such polarization, which produced unobvious effects on M1 markers, but could suppress the levels of M2 markers. GAN could inhibit the phosphorylated expression of STAT6, and M2 macrophages treated by it had a weakened ability to promote malignant behavior of MFC. According to the results of *in vitro* experiments, GAN could inhibit tumor growth, suppress the tissue infiltration of CD206 cells, and inhibit the phosphorylated expression of STAT6.

Conclusion: Our results show that GAN can inhibit the M2 macrophage polarization in gastric cancer microenvironment, whose mechanism of action is associated with the regulation of STAT6 phosphorylation.

## INTRODUCTION

Gastric cancer is a common malignancy with high mortality. Various components in tumor microenvironment (TME), which is a crucial factor in tumor progression and prognosis, interact with tumor cells to promote the malignant behaviors of tumors, including clonality, metastasis and invasiveness [1, 2]. Among them, tumor-associated M2 macrophages are one of the major promoters of gastric cancer progression. Studies have found that M2-TAM could regulate the T cell function, inhibit the NK cell activity and suppress the expressions of IFN-γ and TNF-α, thereby promoting immune escape of tumor cells [3, 4]. Polarization of M2-TAM is regulated by multiple signals, with JAK-STAT being one of the foremost signals facilitating the M2-TAM formation [5, 6]. After phosphorylation and activation, JAK1 promotes the differentiation of macrophages towards different directions through STAT1 and STAT6. Phosphorylation of STAT1 can facilitate the macrophage differentiation into M1 phenotype, which has anti-tumor effects and can secrete substantial inflammatory cytokines, including IFN-γ, TNF-α and IL-1β [7]. Meanwhile, STAT6 phosphorylation can promote the macrophage differentiation towards M2-TAM. Thus, how to inhibit the formation of M2-TAM is a significant means for treating gastric cancer [8, 9].

*Ganoderma lucidum* is a fruiting body of Polyporaceae, which contains substantial amounts of polysaccharides, triterpenoids, sterols and proteins [10]. Currently, the anti-tumor effects of *Ganoderma lucidum* extract are well established. Its triterpenoids, polysaccharides and polypeptides can act directly or indirectly on tumor cells to exert anti-tumor effects, including cell cycle arrest and apoptosis induction, which also have potent regulatory effects on immune cells and molecules [11]. Ganodermanontriol (GAN) is a small natural molecule component of *Ganoderma lucidum* [12]. So far, there has been no clear report on the anti-tumor role or mechanism of GAN, nor has there been any report concerning whether GAN produces a regulatory effect on TAM. Hence, this study investigated the role and mechanism of GAN in regulating the gastric cancer TAM, with a view to further clarifying the anti-tumor role and activity of this small molecule component.

## MATERIALS AND METHODS

### Cell grouping and intervention

Mouse mononuclear macrophage lines RAW264.7 (Procell Biotechnology, Wuhan, China) were chosen for study, which were cultured in a three-gas incubator using special media (Procell Biotechnology, Wuhan, China). After the cells grew to logarithmic phase, their viability was assayed by Trypan blue staining. The macrophages were divided into DMSO, IL-4, GAN-L and GAN-H (10, 20 μM) groups. The DMSO group served as the control, while in the IL-4 group, M2 polarization was induced by 10 ng/ml recombinant IL-4 protein (Abcam, USA) [13]. In the GAN groups, the macrophages were pretreated with GAN for 12 h, and then M2 polarization was induced by IL-4.

During further experiments, we co-cultured mouse gastric cancer MFC (Procell Biotechnology, Wuhan, China) with the RAW264.7 macrophages, in order to observe the M2 polarization effect induced by tumor cells. The cells were divided into DMSO, IL-4, MFC, GAN-L and GAN-H (10, 20 μM) groups. In the GAN groups, the cells were pretreated with GAN for 12 h, and then M2 polarization was induced by IL-4.

### ELISA

In cellular experiments, the levels of M1 markers IFN-γ, TNF-α and IL-1β were detected, as well as the levels of M2 markers IL-10, Arg-1 and TGF-β1. After 48 d of cell induction, the media were isolated, centrifuged for 30 min, and then the supernatant was collected for subsequent experiments. The levels of IFN-γ, TNF-α, IL-1β, IL-10, Arg-1 and TGF-β1 in mouse tumor-bearing tissues were determined. The tissues were crushed with sterile scissors, ground into powder in liquid nitrogen, and lysed on ice for 30 min with 1 ml of NP-40 lysate (Beyotime Biotechnology, Shanghai, China). After adjusting the protein concentration, the supernatant was collected for detection. Cytokine levels were assayed as per the instructions of ELISA kit (Jiancheng Bioengineering Institute, Nanjing, China), and calculated by the standard curve method. The results were expressed as pg/ml.

### Western-blotting

For the detection of p-STAT6 expressions in tissues and cells, the tumor tissues were ground in liquid nitrogen, while the cells were washed twice in PBS. The tissue homogenate and cells were lysed on ice for 30 min with 1 ml of NP-40 lysate to extract proteins, and then the protein concentrations were adjusted after collection of supernatants. Protein concentrations were detected by BCA assay, followed by the concentration adjustment and unification. After denaturation, the protein solutions were subjected to SDS-PAGE analysis and transferred onto membranes. The membranes were then blocked with 5% skim milk powder, incubated at 4°C overnight with TBST-diluted monoclonal antibody (Abcam, USA), and further with HRP-IgG (1:2,000 TBST dilution). Thereafter, chemiluminescent immunoassay was performed, and optical density (OD) was analyzed via Image Pro-Plus 6.0.

### Tumor-bearing mouse model and intervention

Thirty 4-weeks-old female nude mice 20–25 g in body weight were chosen for study, which were divided into Control and GAN (10, 20 mg/kg) groups. After wiping the back of their right hind limbs with iodophor, 0.2 ml of cell suspension was injected to make the cell quantity 5 × 10^6^. One week later, nodular tissues appeared at the injection site of mice. The Control mice were given daily intragastric administration of normal saline, while those in the GAN groups were intragastrically administered with 10 and 20 mg/kg GAN, respectively, once daily for 30 d.

### Immunohistochemical (IHC) staining

The expressions of CD206 in mouse tissues were detected. After preparing the tumor tissues into 4-μm frozen sections, they were treated with 0.01 mol/L citrate buffer, microwaved at 98°C for antigen retrieval, treated with 3% hydrogen peroxide, blocked in 2% BSA, and incubated at 4°C using TBST-diluted CD206 monoclonal antibodies (Abcam, USA). Thereafter, the sections were further incubated for 15 min with peroxidase-labeled streptomycin, and then washed thrice in phosphate buffer, visualized with DAB solution and microscopically observed for chromogenic reaction. Finally, the sections were restained with hematoxylin, sealed and observed under upright microscope.

### Statistical methods

All measurement data were expressed as x̄ ± s, and processed using SPSS 17.0. After homogeneity of variance test, two groups of data were compared by two-independent samples *t*-test, while three or more groups of data were analyzed by one-way ANOVA. Subsequent pairwise comparisons between groups were all made by LSD method. All of the above test processes were two-sided, and differences were considered statistically significant when *P* < 0.05.

### Data availability statement

The data that support the findings of this study are available from the corresponding author upon reasonable request.

## RESULTS

### GAN could inhibit IL-4-induced macrophage M2 polarization

**Figure 1:**
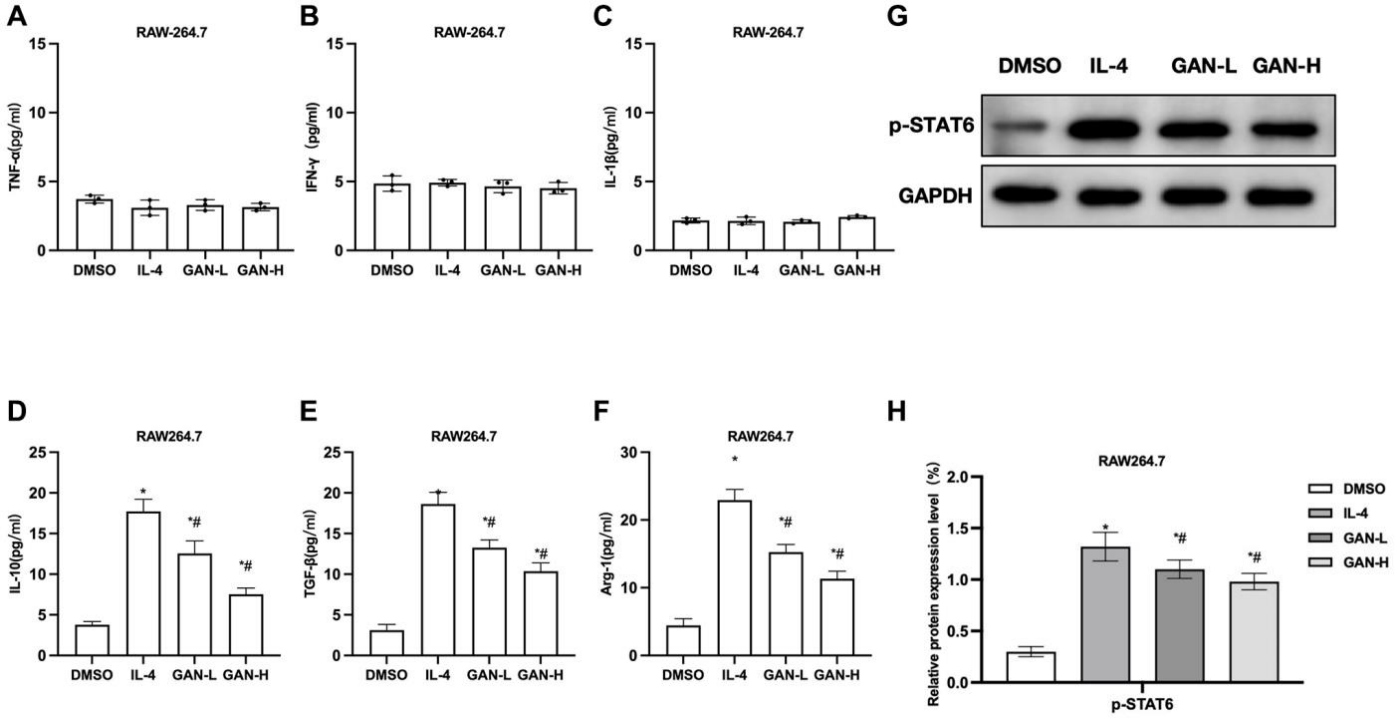
**GAN inhibited the IL-4-induced macrophage M2 polarization.** (**A**–**C**) M1 macrophage markers (*n* = 3). TNF-α, IL-1β and IFN-γ levels in RAW264.7 differed insignificantly from those in J774A.1. (**D**–**F**) M2 macrophage markers (*n* = 3). GAN could inhibit the expressions of IL-10, TGF-β and Arg-1, showing significant differences from the IL-4 group. (**G**, **H**) Relative protein expressions (*n* = 3). Although GAN inhibited the p-STAT6 expression. ^*^P < 0.05 vs. DMSO; ^#^*P* < 0.05 vs. IL-4.

We used IL-4 to induce the M2 activation in two kinds of macrophages. According to the results, IL-4 produced no effect on the expressions of M1 markers TNF-α, IL-1β or IFN-γ, whose levels were low, without showing differences from the Control group. Nonetheless, IL-4 could promote the expressions of M2 markers IL-10, TGF-β and Arg-1, as well as the expression of CD206 and the phosphorylation of STAT6. GAN could inhibit the IL-10, TGF-β, Arg-1 expressions and suppress the STAT6 phosphorylation. Detection of M1 markers revealed that the TNF-α, IL-1β and IFN-γ levels differed insignificantly between the RAW264.7 (Figure 1A–1C). GAN could inhibit the expressions of IL-10, TGF-β and Arg-1, showing significant differences from the IL-4 group (Figure 1D–1F). As suggested by assays of relative protein expressions, GAN inhibited the expression of p-STAT6 (Figure 1G, 1H).

### GAN could inhibit the MFC-induced macrophage M2 polarization

**Figure 2:**
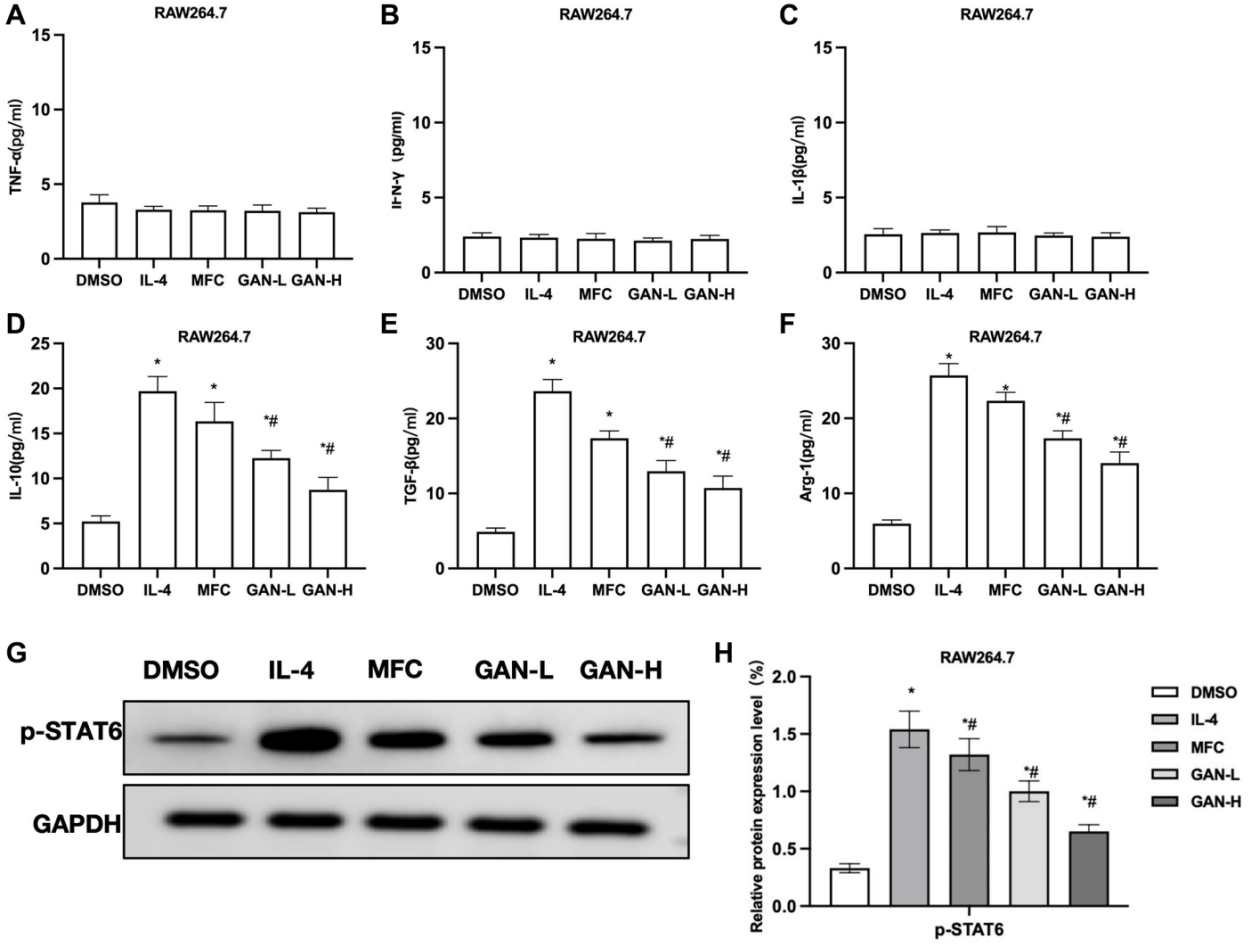
**GAN inhibited macrophage M2 polarization induced by MFC co-culture.** (**A**–**C**) M1 macrophage markers (*n* = 3). MFC co-culture produced unobvious effects on M1 markers, nor did GAN produce any promoting effect on M1 markers, showing insignificant inter-group differences. (**D**–**F**) M2 macrophage marker (*n* = 3). GAN could inhibit the effects of MFC and lower the levels of M2 markers, showing significant differences from the MFC group. (**G**, **H**) Relative protein expressions (*n* = 3). GAN could inhibit the STAT6 phosphorylation and lower the p-STAT6 level. ^*^P < 0.05 vs. DMSO; ^#^*P* < 0.05 vs. IL-4.

MFC co-culture with macrophages could promote the M2 polarization of macrophages, similar to the results induced by IL-4. The MFC co-culture had unobvious effect on M1 markers, nor did GAN produce any promoting effect on M1 markers, showing insignificant inter-group differences (Figure 2A–2C). The MFC-induced M2 polarization could promote the expressions of M2 markers, while GAN could inhibit such effect of MFC and lower the levels of M2 markers, showing significant differences from the MFC group (Figure 2D–2F). As suggested by assays of relative protein expressions, MFC could induce the STAT6 phosphorylation and upregulate the expression of p-STAT6, similar to IL-4. GAN could inhibit the STAT6 phosphorylation and lower the p-STAT6 level (Figure 2G, 2H).

### GAN could inhibit the CD206 invasion in tumors

**Figure 3:**
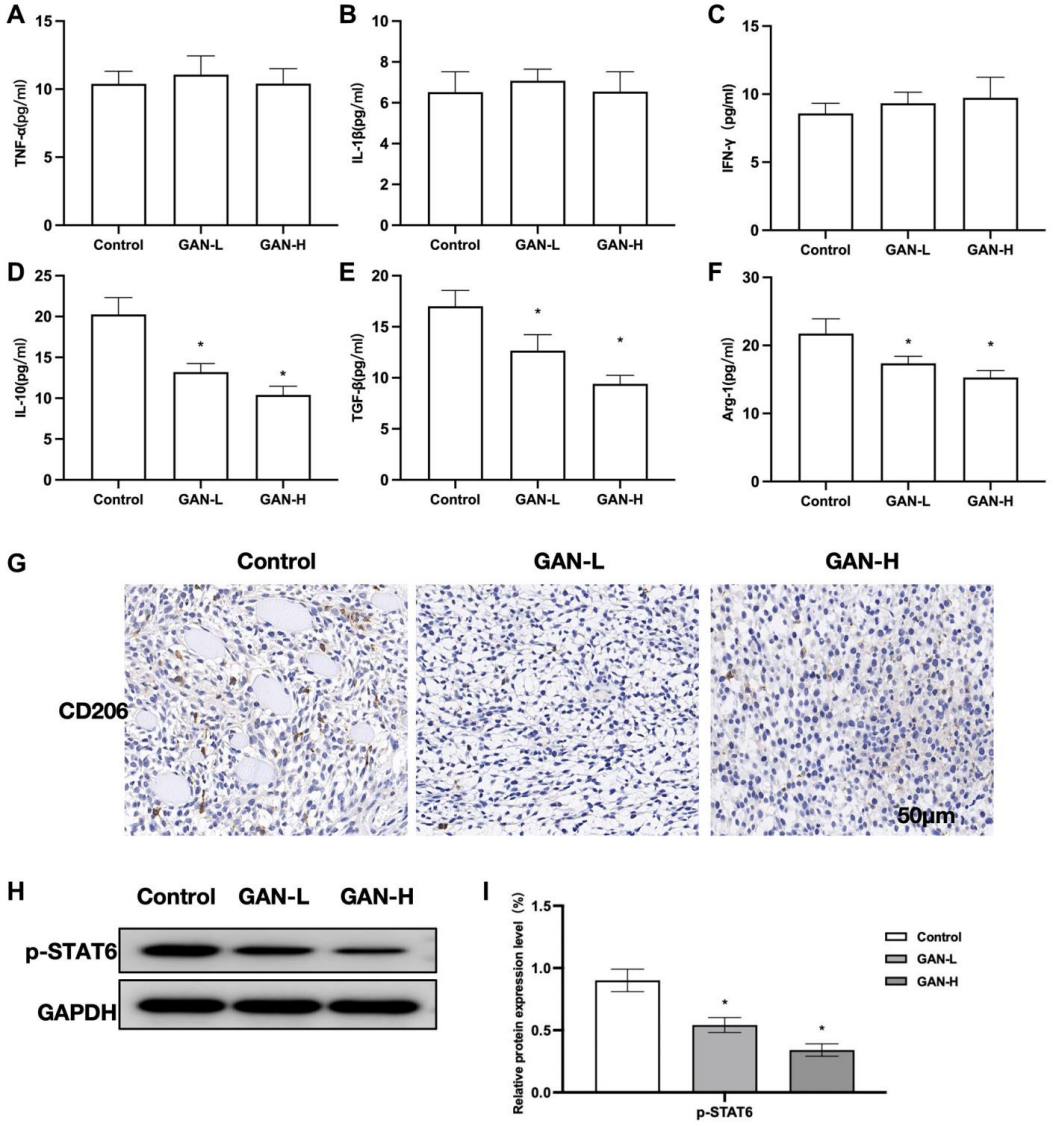
**Effects of GAN on tumor-bearing mice.** (**A**–**C**) M1 markers (*n* = 8). GAN produced unobvious effects on M1 markers, without showing inter-group differences. (**D**–**F**) M2 markers (*n* = 8). GAN could inhibit the expressions of M2 markers, showing significant differences from the Control. (**G**) IHC (*n* = 5). Expressions of CD206 were strongly positive in Control. (**H**, **I**) Relative protein expressions (*n* = 5). GAN could inhibit the phosphorylated expression of STAT6, leading to significantly lower p-STAT6 level than that in Control. ^*^P < 0.05 vs. control.

GAN could inhibit tumor growth and lower the levels of M2 markers in tumor tissues, although producing unobvious effects on M1 markers. It could also reduce the CD206 level. Assays of M1 markers revealed insignificant inter-group differences (Figure 3A–3C). GAN could inhibit the expressions of M2 markers, showing significant differences from Control (Figure 3D–3F). According to the IHC staining results, CD206 was strongly positively expressed in Control, while GAN could inhibit the expressions of CD206. (Figure 3G). Assays of relative protein expressions found that GAN could inhibit the phosphorylated expression of STAT6 (Figure 3H, 3I).

### Binding mode between GAN and STAT6

**Figure 4:**
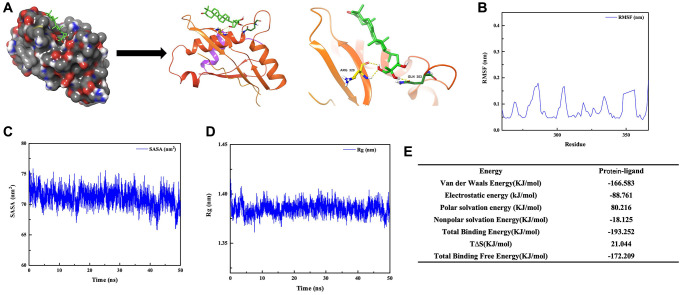
**Binding between GAN and STAT6.** (**A**) Docking result between GAN and STAT6. (**B**–**E**) Molecular dynamics simulations between GAN and STAT6.

Through the small molecule-protein docking model, we found that GAN affected the phosphorylation process of STAT6 by binding to its ARG-324-329 (Figure 4A). Molecular dynamics simulations demonstrated that the free energy was reduced after GAN binding to STAT6, which were bound tightly (Figure 4B–4E).

## DISCUSSION

TAM consists of blood vessels, immune cells, fibroblasts, extracellular matrices, etc. [14], which plays a crucial role in tumorigenesis and development. As the foremost type of immune cells in the microenvironment, TAM originates primarily from myelomonocytes [15]. M2-TAM can secrete IL-10, arginase 1 (Arg-1), programmed death ligand 1 (PD-L1), TGF-β and VEGF, which are conducive to tumor growth [16]. TAM is considered to have the M2 phenotype, a major component of TME [17]. TAM can promote the proliferation, angiogenesis, matrix remodeling and metastasis of tumor cells, and its increase is associated with poor tumor prognosis and short-term survival [18]. Research has shown that high TAM invasion is linked closely to aggressive behavior and associated with abnormal expression of cancer stem cell (CSC) markers, which is an independent prognostic factor for gastric cancer deterioration [19]. Additionally, TAM-mediated chronic inflammation is linked closely to the tumor invasion and metastasis. TAM can also attract Tregs to infiltrate TME by secreting chemokines like CCL4, CCL5, CCL22 and CCL28, thereby preventing the activation of cytotoxic T cells [20]. According to findings of other studies, IL-6 produced by TAM promoted the occurrence of hepatic cancer through STAT3 signaling pathway, while IL-10 produced by TAM promoted the occurrence of non-small cell lung cancer through STATI pathway [21, 22]. Suggestively, TAM-M2 is an important promotor of tumor progression. STATs signals function crucially in the polarization of macrophages, and JAK1 is a vital regulator, whose phosphorylation can promote the activation of STAT1 [23]. Polarization of M1 macrophages is mainly regulated by p-STAT1, which promotes the activation of downstream NLRP3, NF-κB, etc. Meanwhile, a more important role of JAK1 is to promote the activation of STAT6. p-STAT6 is the primary regulatory signal of M2 macrophages, whereas STAT6 is a protein receptor that can promote the M2 polarization of macrophages after phosphorylation at site 324. During the phosphorylation, the levels of Arg-1, TGF-β1, IL-10 and VEGF are upregulated. Suggestively, inhibiting the SATA6 phosphorylation can hinder the effect of JAK1 and inhibit the formation of M2 macrophages [24, 25].

Many existing studies have confirmed that *Ganoderma lucidum*, a kind of fungus, has an excellent anti-tumor activity. Its polysaccharides, triterpenes and peptides all have anti-tumor effects, and the mechanisms are also diverse. GAN, which is a triterpene ketone small molecule, is one major component of *Ganoderma lucidum*. Our results showed that GAN could inhibit the formation of M2-TAM, thereby inhibiting tumor growth. After treatment with IL-4, the main interleukin inducer of M2 polarization, a variety of macrophages were polarized towards M2, as manifested by upregulations of cytokines like Arg-1 and IL-10. Nonetheless, IL-4 had unobvious effects on M1 markers like TNF-α or IFN-γ. Meanwhile, it induced the expression of CD206, which is the foremost marker of M2 macrophages. Pretreatment with GAN could significantly inhibit the M2 polarization of macrophages. Noticeably, GAN failed to affect the expressions of M1 markers, indicating that it did not promote the M1 polarization. GAN merely inhibited the M2 polarization, reducing the expressions of M2 markers like Arg-1 and IL-10. Meanwhile, it also inhibited the expression of CD206. Protein assays revealed that GAN did not affect the expression of STAT6, although producing an obvious effect on p-STAT6, which is the foremost signaling protein that promotes the M2 formation. Inhibition of phosphorylation process can directly affect the M2 formation. We docked GAN with STAT6 and analyzed its molecular dynamics, finding that GAN was bound to ARG-324-329, the phosphorylation site of STAT6. After GAN binding, STAT6 was unable to bind to protein kinase, thus inhibiting the process. This was also proved by the results of dynamic experiments. Finally, in animal experiments, we found that GAN could inhibit the M2 infiltration and polarization, leading to significantly decreased expression of CD206. Meanwhile, GAN could inhibit the expressions of M2 cytokines Arg-1 and IL-10, although producing insignificant effects on M1 markers like TNF-α or IFN-γ. These results verified that GAN influenced M2-TAM, but had an unobvious effect on M1.

## CONCLUSION

Our experimental results show that by binding to STAT6, GAN can regulate the M2 polarization of tumor-associated macrophages after suppressing the STAT6 phosphorylation, thereby inhibiting tumor progression. This is one of the anti-tumor mechanisms of GAN and an important regulatory mechanism for TME immunity, which provides support for its anti-tumor application.
